# Case Report: Unusual electrolyte changes in primary hyperparathyroidism—a call to suspect underlying Gitelman syndrome

**DOI:** 10.3389/fendo.2026.1829964

**Published:** 2026-06-19

**Authors:** Dasili Wickramasinghe Aruna Shantha, Sasmitha Ravindu Waidyatilleke, Kanishka Piumi Senevirathne, Ravindran Dhanushkar, Kushalee Poornima Jayawickreme, Buddika K. Dassanayake, Chamara Dalugama, Manoji Pathirage

**Affiliations:** 1 Department of Medicine, Faculty of Medicine, University of Peradeniya, Peradeniya, Sri Lanka; 2 Department of Surgery, Faculty of Medicine, University of Peradeniya, Peradeniya, Sri Lanka; 3 Department of Endocrinology, Teaching Hospital, Peradeniya, Sri Lanka

**Keywords:** Gitelman syndrome, hypercalcemia, hypomagnesemia, primary hyperparathyroidism, renal tubulopathy

## Abstract

**Background:**

Primary hyperparathyroidism (PHPT) is commonly associated with hypercalcemia, hypophosphatemia, and hypercalciuria. Hypokalemia and hypomagnesemia are rare and indicate a complicated etiology.

**Case presentation:**

We describe a 74-year-old woman who presented with metabolic encephalopathy and moderate dehydration. Biochemical examinations revealed a parathyroid crisis with severe hypercalcemia (4.18 mmol/L), hypophosphatemia, hypokalemic (2.5 mmol/L) metabolic alkalosis, severe hypomagnesemia (0.23 mmol/L), and a high level of intact parathyroid hormone (243.0 pg/mL). The electrolyte imbalances were resistant to proper hydration and electrolyte replacement. Markedly, urine analysis showed renal potassium and magnesium wasting and severe hypocalciuria (calcium-to-creatinine clearance ratio, 0.0093). The presence of this paradoxical hypocalciuria despite PHPT brought into question the possibility of an underlying renal tubulopathy or accompanying vitamin D deficiency. Vitamin D assay showed deficiency with a level of 14.2 ng/mL. Ultrasound neck and contrast-enhanced computed tomography of the neck showed a right thyroid lesion for which the patient underwent right hemithyroidectomy and parathyroidectomy. Histopathological examination revealed a parathyroid adenoma. Although the patient’s hypercalcemia settled postoperatively, hypomagnesemia persisted despite vitamin D supplementation, which indicates the presence of underlying renal tubulopathy, likely Gitelman syndrome.

**Conclusion:**

This case emphasizes that persistent hypokalemia, hypomagnesemia, and unexpected hypocalciuria in patients with PHPT should prompt clinicians to suspect concurrent renal tubulopathies such as Gitelman syndrome.

## 1 Introduction

Primary hyperparathyroidism (PHPT) is a common endocrine disorder caused by autonomous overproduction of the parathyroid hormone (PTH) by one or more parathyroid glands, resulting in prominent electrolyte disturbances, most notably hypercalcemia. Traditionally described by the mnemonic “stones, bones, abdominal groans, and psychic moans”, the clinical manifestation of hypercalcemia has undergone a shift in the developed world over recent decades, with patients being diagnosed at an asymptomatic stage due to widespread routine biochemical screening (1). Nevertheless, where screening is less ubiquitous and in elderly patients whose symptoms are misattributed to age-related changes or comorbidities, PHPT may present as a “parathyroid crisis”. This uncommon and occasionally fatal emergency, which develops in approximately 1%–2% of cases, is defined by severe hypercalcemia (serum calcium >3.5 mmol/L) and acute end-organ dysfunction affecting the central nervous, cardiovascular, and renal systems (2).

PHPT also causes hypophosphatemia and hypercalciuria but does not usually cause hypomagnesemia and hypokalemia (3). Here, we present a case of a patient who presented with a parathyroid crisis along with hypocalciuria and hypomagnesemia resistant to calcium normalization, possibly due to underlying renal tubulopathy, most probably Gitelman syndrome (GS).

## 2 Case description

### 2.1 Clinical presentation

A 74-year-old woman, who was a known patient with dyslipidemia, presented with a history of slurred speech, altered behavior, and memory impairment associated with a mild headache. Her family members attributed her symptoms to a fall she had sustained 3 weeks earlier. Investigations of the patient included a non-contrast computed tomography (NCCT) brain done 1 week prior to admission, which denoted a subacute or chronic lacunar infarction in the right striatocapsular region, and no intra- or extracranial hemorrhages. She had no history of treatment with diuretics, nephrotoxic drugs, alcoholism, or excessive vomiting.

On the day of admission, she was confused with a marked reduction in oral intake. She was unable to communicate effectively due to worsening dysarthria and confusion. There were no reports of fever, overt seizures, or traumatic head injury immediately preceding this acute decline. She complained of constipation, but her urine output was normal, and systemic review revealed no other abnormalities. On examination, she was moderately dehydrated, conscious but disoriented, and her blood pressure was 122/84 mmHg with a pulse rate of 95 bpm and an oxygen saturation of 98%. She had generalized body weakness with intact sensation and no focal neurological signs or meningism. Her other system examination was normal. The absence of focal neurological signs and the presence of significant confusion pointed toward a metabolic encephalopathy.

### 2.2 Diagnostic assessment

Initial investigations (**Table 1**) revealed metabolic alkalosis with severe hypercalcemia, hypokalemia, hypomagnesemia, and hypophosphatemia with normal sodium and creatinine levels. Urine electrolyte studies revealed high urine osmolality, high magnesium fractional excretion (12.5%), a high transtubular potassium gradient of 6, a high fractional excretion of PO_4_^3−^ (22%) with high tubular maximum reabsorption of phosphate-to-glomerular filtration rate ratio of 0.35 mmol/L, but notably low calcium levels with a low creatinine-to-calcium ratio (0.0093). These persistent electrolyte derangements, despite adequate fluid resuscitation and electrolyte replacement, arose suspicion of PHPT or paraneoplastic syndromes causing hyperparathyroidism, with acquired or pre-existing renal tubulopathy (GS) or vitamin D deficiency.

**Table 1:** Initial biochemical investigations.

Investigation	Parameter	Result	Normal range
Full blood count	White blood count (10^3^/µL)	12.10	4.00–10.00
	Hemoglobin (g/dL)	13.4	11.00–15.00
	Platelet count (10^3^/µL)	356	150–450
CRP		4.1	0.0–5.0
Serum electrolytes (mmol/L)	Sodium	133	136–145
	Potassium	2.5	3.5–5.1
	Corrected calcium	4.18	2.10–2.50
	Magnesium	0.23	0.73–1.06
	Phosphorus	0.45	0.81–1.45
Osmolality (mOsm/kg)	Serum osmolality	293	235–275
	Urine osmolality	464	
Serum creatinine (μmol/L)		41.74	49.0–115.0
Urine electrolytes (mmol/L)	Sodium	89.6	<10
	Potassium	76.2	<10
Urine electrolyte studies	Magnesium fractional excretion (%)	12.53	<4
	Phosphate fractional excretion (%)	22.45	<20
	TmP/GFR (mmol/L)	0.35	0.70–1.35
	Calcium-to-creatinine clearance ratio (mg/mg)	0.0093	0.01–0.25
	Transtubular potassium gradient	6	<3 (in hypokalemia)
Intact PTH (pg/mL)		243.0	8.7–79.6
Vitamin D (ng/mL)		14.2	30 – 50

CRP, C-reactive protein; TmP/GFR, tubular maximum phosphate reabsorption per glomerular filtration rate; PTH, parathyroid hormone.

**Figure 1:**
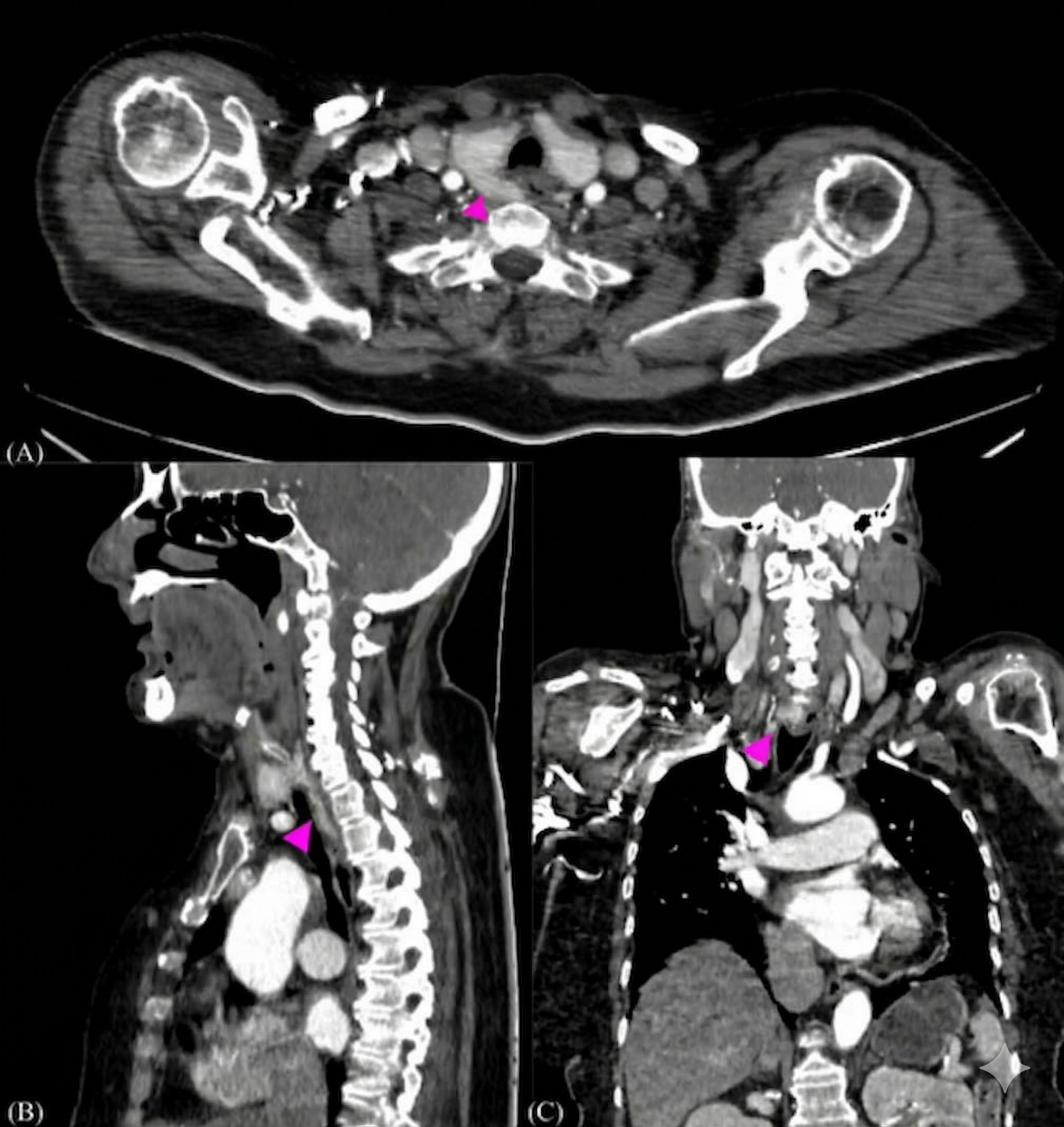
Contrast-enhanced computed tomography of the right thyroid lesion (purple pointer). **(A)** Transverse view. **(B)** Sagittal view. **(C)** Coronal view.

Serum vitamin D assay revealed severe vitamin D deficiency and intact PTH revealed overt hyperparathyroidism. Ultrasound scan of the neck revealed a thyroid gland with coarse echotexture and a hyperechoic vague exophytic nodule with no increased vascularity measuring 6.7 mm × 5.4 mm × 9.3 mm in the right lower pole of the thyroid queried to be a right lower pole thyroid adenoma or parathyroid, with a suggestion to perform a 4D-CT to exclude the latter. However, the limitation of resources prevented a 4D-CT or Sestamibi uptake scan; thus, a contrast-enhanced CT of neck, chest, abdomen, and pelvis was performed, revealing an elongated mass extending from the inferior pole of the right lobe of the thyroid medially, inferiorly, and in the retro-tracheal space for a length of 3 cm, with no cervical or mediastinal lymphadenopathy (**Figure 1**).

A multidisciplinary meeting decided to formulate a diagnosis of PHPT caused by parathyroid adenoma complicated with hypercalcemia and coexisting vitamin D deficiency with possible GS, and to proceed with surgery.

### 2.3 Therapeutic interventions

Electrolyte correction was done with intravenous and oral potassium chloride supplementation alongside intravenous and oral magnesium replacement. However, the electrolyte derangements were persistent. Intravenous zoledronic acid was administered to manage her hypercalcemia, and the patient was supplemented with 100,000 IU of vitamin D preoperatively to prevent hungry bone syndrome after parathyroidectomy. The patient underwent right hemithyroidectomy, parathyroidectomy, and lymph node excision, after which her symptoms and electrolyte derangements greatly improved. Histopathology revealed the appearance of parathyroid adenoma with a reactive lymph node.

### 2.4 Follow-up and outcome

**Figure 2:**
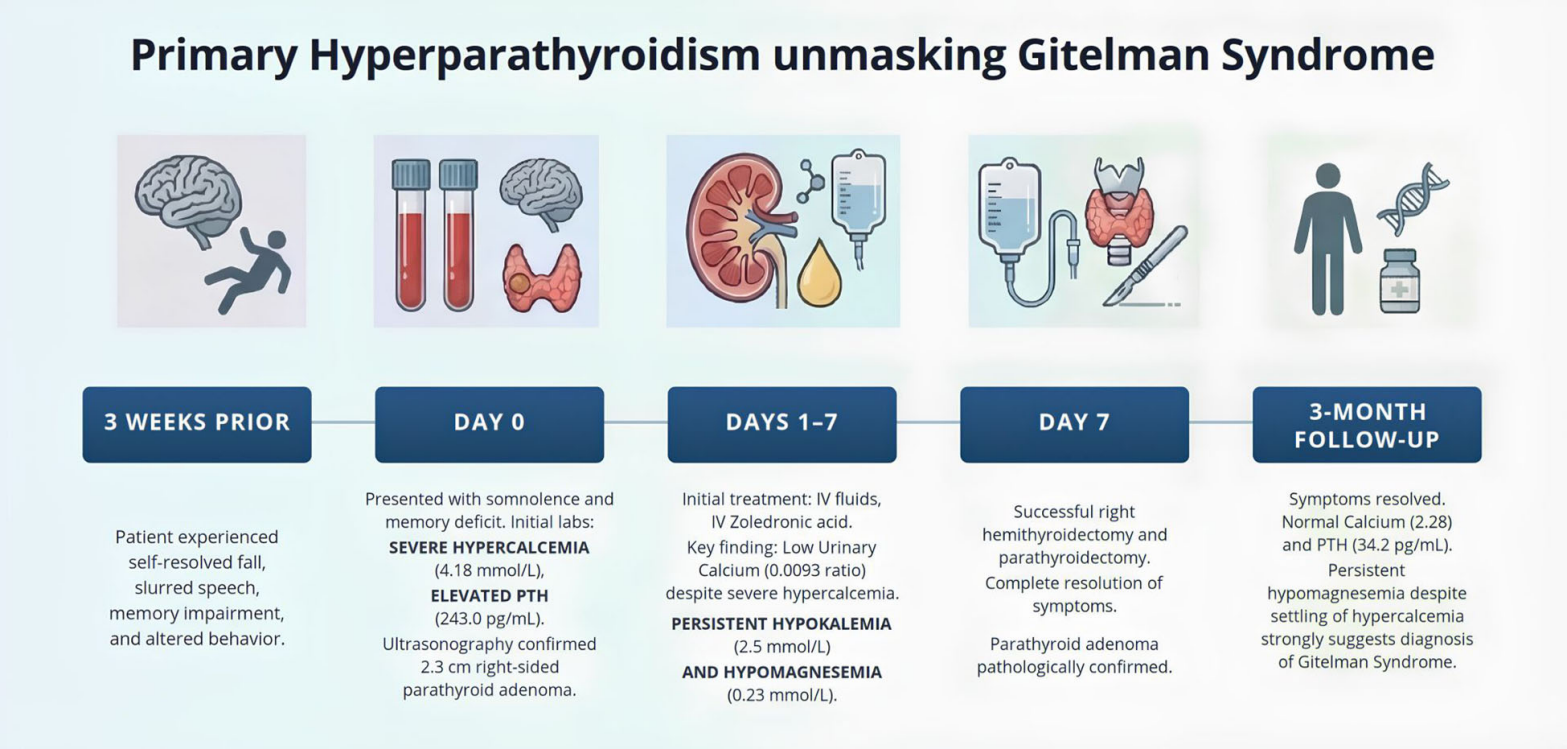
Timeline of the clinical progression of this case.

On the third month of follow-up, she is on daily spironolactone therapy, along with magnesium, potassium, and vitamin D supplementation. She is symptom-free but remains hypomagnesemic, which favors the hypothesis of pre-existing GS being the cause of the electrolyte derangements over vitamin D deficiency, as vitamin D levels typically reach normal limits within 8–12 weeks of initiation of supplementation (4). Resource limitation prevents genetic testing for definitive diagnosis of GS (**Figure 2**).

## 3 Discussion

This case denotes a complex, multisystemic presentation of PHPT. Rather than an “asymptomatic” biochemical finding, her condition manifested as a life-threatening endocrine emergency characterized by metabolic encephalopathy. The pathophysiological mechanisms of the unusual electrolyte disturbances—hypomagnesemia and hypokalemia—caused by the underlying renal tubulopathy are explained in the following discussion with the aid of the current literature.

PHPT is characterized by electrolyte derangements due to excessive PTH secretion. The defining feature is hypercalcemia, resulting from PTH-mediated increased bone resorption, enhanced renal reabsorption of calcium in the distal tubules, and elevated intestinal calcium absorption (3). Although PTH increases calcium reabsorption, the vast magnitude of filtered calcium due to hypercalcemia surpasses the threshold of this mechanism, causing hypercalciuria (5). Furthermore, PHPT typically also causes hypophosphatemia, as PTH promotes renal excretion of phosphate (3). While hypercalcemia and hypophosphatemia are hallmarks of PHPT, hypokalemia is observed in some patients, often linked to hypercalcemia activating the calcium-sensing receptor in the thick ascending limb of Henle, which inactivates the sodium-potassium-2 chloride cotransporter and can induce hypokalemic metabolic alkalosis, mimicking the effects of loop diuretics (6). Secondary hyperaldosteronism due to intravascular volume depletion can further exacerbate hypokalemia (6). Hypomagnesemia may very rarely be present in PHPT, and its presence is associated with a more severe presentation of the disease, often alongside higher serum calcium and PTH levels (7). Whether it is a cause or effect of PHPT is still unclear. These electrolyte disturbances contribute to the clinical manifestations seen in PHPT. In our patient, the continued hypocalciuria with hypercalcemia raised suspicion of underlying vitamin D deficiency or GS, which was reinforced by severe hypomagnesemia and persistent hypokalemia despite correction of hypercalcemia. Although familial hypocalciuric hypercalcemia also causes the combination of its namesake, the absence of the usually associated hypermagnesemia led us to exclude it from our differentials (8). The patient had not been treated with any diuretics, and although she did have some vomiting in the initial phase of illness, it was not voluminous enough to cause the persistently low potassium and magnesium levels, which led us to entertain the possibility of an underlying tubulopathy like GS.

GS is an autosomal recessive renal tubulopathy primarily caused by inactivating mutations in the *SLC12A3* gene, which encodes the thiazide-sensitive sodium-chloride cotransporter (NCC) located in the apical membrane of the distal convoluted tubule, and is characterized by chronic hypokalemic metabolic alkalosis, hypomagnesemia, and hypocalciuria (9, 10). This defective NCC function leads to impaired reabsorption of sodium and chloride, causing renal salt wasting and subsequent volume contraction (10). The high sodium load stimulates its uptake via ENaC channels, which drives the secretion of potassium and hydrogen. Furthermore, volume depletion due to NCC dysfunction activates the renin–angiotensin–aldosterone system, leading to secondary hyperaldosteronism. This, exacerbates the loss of potassium and hydrogen ions in the collecting tubule, contributing to characteristic hypokalemia and metabolic alkalosis (10). GS is also marked by hypomagnesemia due to reduced expression or dysfunction of TRPM6 magnesium channels in both the distal convoluted tubule and the intestine (11). Hypocalciuria, which prompted us to consider renal tubulopathies and vitamin D deficiency in our list of differentials complicating hyperparathyroidism, is also a hallmark of GS, resulting from increased passive calcium reabsorption in the proximal convoluted tubule (10). This increased capacity for reabsorption of calcium may be substantive enough to reabsorb the increased load of filtered calcium caused by PHPT, which would otherwise cause hypercalciuria. Furthermore, patients with GS exhibit hypomagnesemia-induced PTH secretion dysfunction and end-organ resistance to PTH, effectively protecting them from hypercalcemia due to increased renal reabsorption caused by PHPT (12). However, the co-existing PHPT due to parathyroid adenoma in our patient caused her to be hypercalcemic. Resource scarcity prevented *SLC12A3* gene testing, which would have confirmed our clinical diagnosis of GS.

There have been several reports of unusual electrolyte abnormalities in patients with PHPT (**Table 2**), only five being due to underlying GS. Our case represents the sixth known case of co-occurrence of PHPT and GS, which might point toward an association between the two conditions, which warrants the need for further large-scale research.

**Table 2:** Summary of published cases of primary hyperparathyroidism with unusual electrolyte derangements.

Authors, year	Clinical presentation	Electrolyte and pH abnormalities	Final diagnosis	Management	Outcome
Menghua Yuan, et al., 2025 (5)	Recurrent, severe hypercalcemic crises	↑ - Ca^2+^, PTH, urine Mg^2+^ (mild) ↓ - PO_4_^3+^, K^+^, Mg^2+^, urine Ca^2+^	PHPT due to parathyroid adenoma, concomitant with GS, confirmed by postoperative pathology and genetic testing for SLC12A3 mutation.	Treatment of hypercalcemia with fluid resuscitation, calcitonin, zoledronic acid, and denosumab. Vitamin D, Mg, and K supplementation, and parathyroidectomy.	No specific mention.
Shanshen Yu, Jia Sun, Lijun Mou, 2024 (9)	46-year-old female patient with >10-year history of T2DM, 12-year history of ↓ - K^+^, Mg^2+^ Intermittent joint swelling and pain.	↑ - Ca^2+^, PTH, urine K^+^, urine Mg^2+^ (mild) ↓ - K^+^, Mg^2+^, urine Ca^2+^ Metabolic alkalosis	GS concomitant with PHPT. GS confirmed by homozygous mutation in SLC12A3 (c.179C > T [p.T60M]). PHPT consistent with left parathyroid hyperplasia on ultrasound.	Potassium and magnesium supplementation.	Serum electrolyte levels normalized and joint pain was relieved.
Teresa Rego, Fernando Fonseca, Rita Cerqueira, Ana Agapito, 2018 (12)	25-year-old female patient with malaise, fatigue, myalgias, cramps, left hemiface and left upper arm paraesthesias.	↑ - Ca^2+^, PTH, urine Mg^2+^ ↓ - K^+^, Mg^2+^, PO_4_^3−^ Metabolic alkalosis	GS coexisting with PHPT due to parathyroid adenoma. GS - heterozygous mutations (c.602-16G>A and c.2221G>A (p.Gly741Arg) in SLC12A3 gene.	Inferior left parathyroidectomy. Lifelong treatment with spironolactone.	Persistent mild to moderate hypomagnesemia. All other electrolytes normalized.
Zeinab Alnahas, Marko Markov, Mohamad H. Horani, 2022 (13)	36-year-old man with bipolar depression disorder, GERD, and AF presenting after a motor accident.	↑ - Ca^2+^ (mild), PTH, urinary K^+^ ↓ - K^+^, Mg^2+^, PO_4_^3−^ (mild), urinary Ca^2+^	PHPT due to parathyroid adenoma with concomitant GS.	Inferior left parathyroidectomy.	Resolution of increased serum calcium and PTH levels.
Yao-Ko Wen, 2011 (14)	41-year-old known female patient with past and family history of GS, presenting with incidentally detected ↑Ca^2+^ for 2 years.	↑ - Ca^2+^ (mild) ↓ - K^+^, Mg^2+^, PO_4_^3−^ (mild), 25-hydroxyvitamin D, urinary Ca^2+^	PHPT with coexisting GS. PHPT was diagnosed due to normal PTH levels despite ↑Ca^2+^ levels and histological confirmation of parathyroid adenoma.	Classic neck exploration with a horizontal thyroid incision	Normalization of serum calcium levels.
Tom Edward Ngo Lo, Iris Thiele Isip Tan, 2015 (15)	A 26-year-old Filipino man presenting with recurrent nephrolithiasis, leading to distal renal tubular acidosis manifesting with hypokalemic periodic paralysis and hypomagnesemia.	↑ - Ca^2+^, PTH, Cl^-^, serum creatine, urine Ca^2+^ ↓ - K^+^, Mg^2+^, urine Ca^2+^ Normal anion gap metabolic acidosis, urine alkalosis	Distal renal tubular acidosis due to primary hyperparathyroidism, caused by a solitary functioning parathyroid adenoma. Concomitant distal RTA was diagnosed based on the absence of proteinuria, glucosuria, alkaline urine pH, and inability to acidify urine despite acidosis.	Hypercalcemia managed with intravenous hydration and increased oral fluid intake. Minimally invasive selective parathyroidectomy was performed.	Complete reversal of ↑Ca^2+^ and ↑PTH parathyroidectomy
A. A. Siddiqui, D. R. Wilson, 1972 (16)	Two patients presenting with renal calculi. Case 1—21-year-old slim-built female patient, Case 2—35-year-old female patient with obesity	↑ - Ca^2+^, PTH, Cl^-^, serum creatine, urine Ca^2+^ ↓ - PO_4_^3+^, HCO_3_^-^, K^+^ Case 1—increased urinary hydroxyproline excretion suggesting increased bone turnover.	Primary hyperparathyroidism due to a parathyroid adenoma with proximal (bicarbonate-wasting) renal tubular acidosis.	Parathyroid exploration and removal of a solitary adenoma in each case	Case 1—developed hypocalcemia post-operatively, which was treated with supplementation and discontinued later with settling of hypocalcemia. Case 2—↑Cl ^–^ and ↓HCO_3_^-^ persisted up to 12 months postoperatively.
Jayaraman Muthukrishnan, K. et al., 2008 (17)	Four patients with bone pains, nephrocalcinosis, nephrolithiasis and metabolic myopathy. One patient presented with recurrent renal stones.	Alkaline fasting urine (pH >5.5) Normal anion gap metabolic acidosis Hypercalciuria	Distal renal tubular acidosis in patients with symptomatic primary hyperparathyroidism, confirmed by an ammonium chloride loading test.	Surgical cure of PHPT by removal of parathyroid adenoma.	Complete resolution of distal RTA after surgical cure of PHPT, with normalization of the acid-base status in three patients.
Paul Fourman, Brian McConkey, J. W.G. Smith, 1960 (18)	This work describes a study of 6 patients with hyperparathyroidism without polyuria. Clinical findings included bone disease and renal stones. One patient had multiple parathyroid and islet cell tumors with hypoglycemia. Another had a solitary osteoclastoma.	↑ - Ca^2+^, PTH Metabolic acidosis Urinary alkalosis	Renal tubular acidosis in patients with hyperparathyroidism.	All patients with hyperparathyroidism had their parathyroid adenomas removed.	Not detailed as specific case outcomes.
James W. Agna, Richard E. Goldsmith, 1958 (19)	Three patients with primary hyperparathyroidism. Case 1—32-year-old woman with symptoms of hypercalcemia, gastroenteric disease, and a renal calculus. Case 2—8-year-old boy with joint pain, fever, lethargy, and generalized non-rhythmical twitching. Case 3—37-year-old woman with generalized hyper-reflexia despite concurrent hypokalemia and hypercalcemia.	↑ - Ca^2+^, PTH ↓ - Mg^2+^, K^+^	Primary hyperparathyroidism associated with hypomagnesemia, confirmed by surgical exploration for adenomatous hyperparathyroidism	Parathyroid surgery was performed on all patients. Magnesium sulfate was administered intramuscularly to Case 1 and Case 2 to treat symptoms of magnesium deficiency. Case 3 did not receive magnesium therapy.	Complete recovery in all cases.

PHPT, primary hyperparathyroidism; GS, Gitelman syndrome; GERD, gastro-esophageal reflux disease; AF, atrial fibrillation. Upwards and downwards arrows as “Increased concentrations” and “Decreased concentrations” respectively. ↑, Increased concnetrations; ↓, Decreased concentrations.

## 4 Conclusions

Although PHPT is known to cause electrolyte derangements, persistent unusual abnormalities like hypokalemia, hypomagnesemia, and hypocalciuria warrant investigation for pre-existing or acquired, coexisting renal tubulopathies.

## 5 Patient perspective

I am 74 years old. A few weeks before going to the hospital, I became very weak, my speech slurred, and I was very confused. My family and I thought it was just from a recent fall. By the time I was admitted, I could barely talk.

The doctors told me that my blood salts were very wrong and that there is a small lump in my neck. After giving me some medicines, they did surgery to remove the lump. Within a few days, my mind cleared and my strength came back! Now I feel completely fine. I just have to take a daily magnesium pill. I am very thankful to the doctors for giving me my life back.

## Data Availability

The original contributions presented in the study are included in the article/supplementary material. Further inquiries can be directed to the corresponding author.
